# Long–term lung inflammation is reduced by estradiol treatment in brain dead female rats

**DOI:** 10.6061/clinics/2021/e3042

**Published:** 2021-08-05

**Authors:** Fernanda Yamamoto Ricardo-da-Silva, Roberto Armstrong-Jr, Marina Vidal-dos-Santos, Cristiano de Jesus Correia, Raphael dos Santos Coutinho e Silva, Lucas Ferreira da Anunciação, Luiz Felipe Pinho Moreira, Henri Gerrit Derk Leuvenink, Ana Cristina Breithaupt-Faloppa

**Affiliations:** I Laboratorio de Cirurgia Cardiovascular e Fisiopatologia da Circulacao (LIM-11), Instituto do Coracao (InCor), Hospital das Clinicas HCFMUSP, Faculdade de Medicina, Universidade de Sao Paulo, Sao Paulo, SP, BR; II Department of Surgery, University Medical Centre Groningen, University of Groningen, The Netherlands

**Keywords:** Brain Death, Organ Donor, Lung Inflammation, Estradiol, Female Rats

## Abstract

**OBJECTIVES:**

Lung transplantation is limited by the systemic repercussions of brain death (BD). Studies have shown the potential protective role of 17β-estradiol on the lungs. Here, we aimed to investigate the effect of estradiol on the long-lasting lung inflammatory state to understand a possible therapeutic application in lung donors with BD.

**METHODS:**

Female Wistar rats were separated into 3 groups: BD, subjected to brain death (6h); E2-T0, treated with 17β-estradiol (50 μg/mL, 2 mL/h) immediately after brain death; and E2-T3, treated with 17β-estradiol (50 μg/ml, 2 ml/h) after 3h of BD. Complement system activity and macrophage presence were analyzed. TNF-α, IL-1β, IL-10, and IL-6 gene expression (RT-PCR) and levels in 24h lung culture medium were quantified. Finally, analysis of caspase-3 gene and protein expression in the lung was performed.

**RESULTS:**

Estradiol reduced complement C3 protein and gene expression. The presence of lung macrophages was not modified by estradiol, but the release of inflammatory mediators was reduced and TNF-α and IL-1β gene expression were reduced in the E2-T3 group. In addition, caspase-3 protein expression was reduced by estradiol in the same group.

**CONCLUSIONS:**

Brain death-induced lung inflammation in females is modulated by estradiol treatment. Study data suggest that estradiol can control the inflammatory response by modulating the release of mediators after brain death in the long term. These results strengthen the idea of estradiol as a therapy for donor lungs and improving transplant outcomes.

## INTRODUCTION

Lung transplantation is the main treatment option for patients with end-stage lung failure. However, despite advancements in the medical field, there is still a shortage of suitable lungs for transplantation (1). Although they mainly originate from brain dead donors (2), brain death (BD) itself causes hemodynamic, hormonal, and inflammatory effects, which left alone, could decrease long-term transplant survival (3,4). An often-neglected aspect of the effects of BD in lung grafts is the immune response difference in female and male donors. Studies show that hormonally active women tolerate trauma better than men, producing less cytokines and having lower mortality (5,6). However, in lung transplantation, there is a higher risk of mortality for male recipients with grafts from female donors (7,8). Hormonally active women, who would normally have protection from traumatic injuries, could be considered worse donors if BD results in a decline in female sex hormones. This is illustrated by experimental studies comparing brain dead female and male rats, where more severe lung injury in females was associated with the reduction of female sex hormones, especially estradiol (9-11). Thus, understanding the long-term role of estradiol in female lung donors can contribute to therapeutic donor management aiming to improve transplant outcomes. In this sense, the evaluation of estradiol’s influence on the complement system activity, which increases BD-induced lung injury and worsens transplant outcomes (12,13), as well as on the inflammatory response with activated neutrophil and macrophage release of inflammatory mediators, is relevant. Therefore, in this study, we investigated the effect of estradiol on BD-induced lung inflammation, focusing on the long-term inflammatory repercussions and complementary elements as a means to understand estradiol as a possible therapeutic tool for lung transplantation.

## MATERIALS AND METHODS

### Animals

Our study used female Wistar rats (60 days, n=32) during the estrus or proestrus phases of the estral cycle (identified via vaginal smears). The animals were housed at 23±2°C, with a cycle of 12-h light-dark and *ad libitum* food and water. All experiments were conducted according to the humane care established by the “Principles of Laboratory Animal Care” written by the National Society for Medical Research and the “Guide for the Care and Use of Laboratory Animals” written by the Institute of Laboratory Animal Resources, published by the National Institute of Health (NIH Publication N°86-23, revised 1996). Experiments in this study were also approved by the Faculdade de Medicina da Universidade de São Paulo Ethic Committee for Research Projects Analysis (SDC N°4350/16/016).

### Groups and treatment

Animals were randomly divided into three groups: BD, animals subjected to BD; E2-T0, animals subjected to BD and treated with estradiol for 6h, starting after BD confirmation (T0); and E2-T3, animals subjected to BD and treated with estradiol for 3h, starting 3h after BD confirmation (T3). The animals from the treated groups received an intravenous infusion of 17β-estradiol (E2-50 μg/mL, Sigma-Aldrich^®^, USA) diluted in saline solution and administered continuously (2 mL/h). For reference values, a group of false operated (sham) animals were subjected to cranial perforation without a catheter and maintained for 6h under anesthesia and mechanical ventilation.

### Experimental brain death model

BD was induced as described by Breithaupt-Faloppa et al. (9). Animals were anesthetized with isoflurane, and the carotid artery and jugular vein were catheterized for the mean arterial pressure (MAP) measurement and fluid replacement and/or treatment administration. A balloon catheter (Fogarty-4F, Baxter Healthcare Co., USA) was inserted into the intracranial space and BD was induced by rapid inflation with 400 μL saline solution into the balloon. After BD confirmation by increased MAP, reflex absence, and fixed and maximally dilated pupil, anesthesia was interrupted. All animals were monitored for 6h during BD.

### Serum C3 quantification

The complement system component C3 was measured using a commercial kit in serum samples, following the manufacturer’s instructions (Abcam, UK).

### Immunohistochemistry

The left lung lobe was insufflated with diluted Tissue-Tek O.C.T. Compound (1:3, Sakura Finitek, Japan) and frozen in a hexane solution using nitrogen. Cryosections of 10 μm were fixed with acetone on a glass slide. For the immunohistochemistry reaction, endogenous peroxidase was blocked (H_2_O_2_, 2%) followed by unspecific site blockage with bovine serum albumin/Tris-buffered saline – Tween 20 (1%). Subsequently, the slices were incubated with the primary antibodies (1h at 37°C): anti-C5b-9 (1:200; Hycult Biotech, The Netherlands), anti-CD80 (1:100 - Abcam), anti-C3 (1:100; Millipore Corporation, USA), and anti-caspase-3 (1:100 - Abcam). Secondary antibodies conjugated with horseradish peroxidase (HRP) were incubated for 1h at 37°C: C5b-9 with anti-mouse at 1:200 (Millipore Corporation), CD80 with anti-rabbit at 1:200 (Millipore Corporation), C3 with anti-goat at 1:200 (Santa Cruz Biotechnology, USA), and caspase-3 with anti-rabbit at 1:200 (Millipore Corporation). Staining was performed using HRP as substrate (Diaminobenzidine, Dako, USA). As a negative control, we incubated sections in the absence of the first antibody. Staining areas were quantified in lung sections after determining the threshold and staining area fraction with an image analyzer (NIS Elements, Nikon, Japan).

To analyze the CD163 expression, lung tissue samples were embedded in paraffin and 4-μm sections were cut. After deparaffinization, antigen retrieval (TRIS/HCl (pH 9.0) overnight, 80°C) and endogenous peroxidase blocking (H_2_O_2_, 2%) were performed. The CD163 primary antibody (Abcam) was incubated at 1:500, the secondary antibody conjugated to HRP (goat-anti-rabbit/HRP, Dako, USA) at 1:100, and the secondary antibody (rabbit-anti-goat/HRP, Dako, USA) at 1:100. Finally, the reaction was developed by incubation with diaminobenzidine (Dako, USA). As a negative control, we incubated sections in the absence of the first antibody. Staining areas were quantified in lung sections after determining the threshold and staining area fraction with an image analyzer (NIS Elements, Nikon, Japan).

### Gene expression analysis

Lung samples were stored at -80°C in an RNA stabilization solution. RNA was extracted with a commercial kit (mirVana Kit, Ambion, USA) and cDNA was transcribed using a High-Capacity Reverse Transcriptase Kit (Applied Biosystems, USA). Real-time polymerase chain reaction (PCR) was performed with SYBR^®^Green PCR Master Mix (Applied Biosystems, USA) and SYBR^®^Green primers (Applied Biosystems, USA) against β-actin, C3, C3aR, decay-accelerating factor (DAF), tumor necrosis factor (TNF)-α, interleukin (IL)-1β, IL-10, IL-6, and Caspase-3 (Table 1). Targets were amplified over one cycle for 2 min at 50°C, one cycle for 10 min at 95°C, 40 cycles for 15s at 95°C, and for 1 min at 60°C. Relative gene expression was determined in relation to reference values obtained from sham animals (n=5).

### Lung *ex vivo* culture

Following Breithaupt-Faloppa et al. (14), the lungs were perfused through the pulmonary artery. The lung was then cut into 4 small fragments, placed in 1 mL of Dulbecco’s modified Eagle medium (Vitrocell, Brazil) at 37°C in a humidified atmosphere of 5% CO_2_ for 24h. At the end of the incubation period, lung fragments were dried and weighed, and the medium was stored at -80°C for further analysis.

### Lung culture inflammatory mediator quantification

TNF-α and IL-1β were quantified in lung culture medium with enzyme-linked immunosorbent assay (ELISA) kits performed according to the manufacturer’s instructions (R&D Systems, Inc., USA). IL-10 and IL-6 were also quantified in lung culture medium, but using a Milliplex MAP kit (EMD Millipore Corporation, USA) using a Luminex 200 system with xPonent Analyst Software v.4.2 (EMD Millipore Corporation). The data were expressed as pg/ml/mg dry weight.

### Statistical analysis

Study data are presented as means±standard error of the mean (SEM) or median and 95% percentile interval (all gene expression data). Comparisons between groups of data were performed using GraphPad Prism Software v.8.3.1 (GraphPad Software Inc, USA), using the Kruskal-Wallis test followed by Dunńs test for multiple comparisons.

## RESULTS

### Estradiol’s effect on complement system response to BD

**Figure 1:**
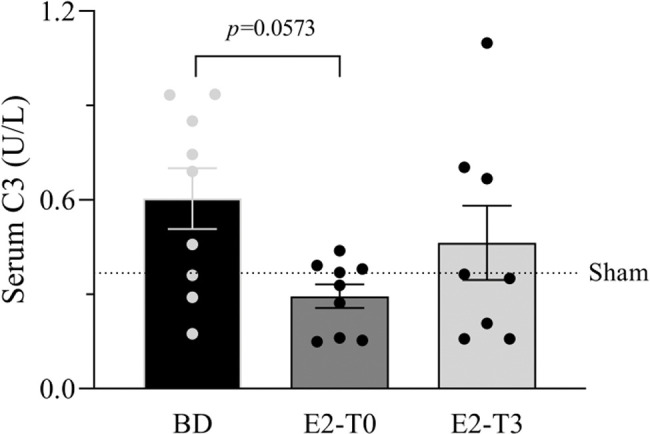
Serum quantification of complement system C3 component. Female rats were separated in groups: sham animals as control, BD animals subjected to brain death (BD), E2-T0 animals subjected to BD and treated with 17β-estradiol (E2) starting after BD confirmation, and E2-T3 animals subjected to BD and treated with E2 3h after BD confirmation. Data are expressed as mean±SEM from 8-9 animals. Kruskal-Wallis *p*=0.0891.

**Figure 2:**
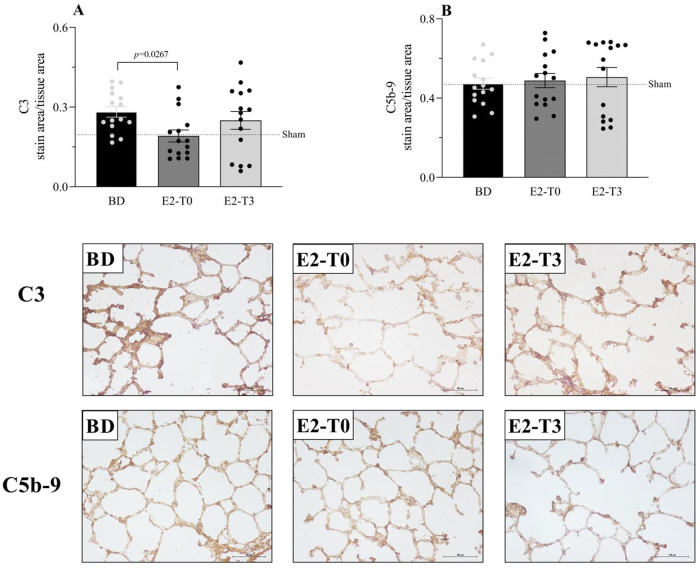
Complement system C3 (A) and C5b-9 (B) component expression on lung tissue and respective photomicrography of the immunohistochemical reaction. Female rats were separated in groups: sham animals as control, BD animals subjected to brain death (BD), E2-T0 animals subjected to BD and treated with 17β-estradiol (E2) starting after BD confirmation, and E2-T3 animals subjected to BD and treated with E2 3h after BD confirmation. Data expressed as mean±SEM from 5 photos/sample, 3 samples/animal, 5 animals/group. Kruskal-Wallis *p*=0.0442 (A) and *p*=0.8881 (B).

**Figure 3:**
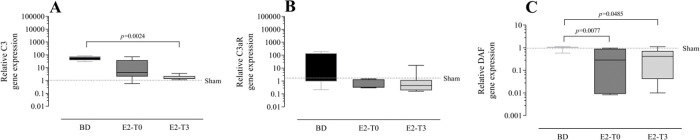
Lung gene expression of complement system components C3 (A), C3aR (B) and decay-accelerating factor (DAF) (C). Female rats were separated in groups: sham animals as control, BD animals subjected to brain death (BD), E2-T0 animals subjected to BD and treated with 17β-estradiol (E2) starting after BD confirmation, and E2-T3 animals subjected to BD and treated with E2 3h after BD confirmation. Data are expressed as median and 95% percentile interval from 6 animals. Kruskal-Wallis *p*=0.0015 (A), *p*=0.1715 (B) and *p*=0.005 (C).

Figure 1 presents the serum quantification of the complement system C3 components. The high concentration of C3 in animals from the BD group was reduced by the longer estradiol treatment (E2-T0). The C3 deposit in the lung tissue is shown by immunohistochemistry analysis in Figure 2A. The expression of C3 in the lung tissue was also reduced by treatment with E2 for 6h (E2-T0). On the other hand, the C5b9 deposit in the lung tissue was not influenced by E2 treatment (Figure 2B). In addition, we analyzed the gene expression of the complement system components (Figure 3). Genes for the C3 and C3a receptor (C3aR) were upregulated after BD, and estradiol treatment reduced C3 and downregulated C3aR, especially in the E2-T3 group (Figure 3 A, B). The DAF gene expression was not altered by BD; however, estradiol treatment had a downregulatory effect.

### Macrophage presence in lung tissue

**Figure 4:**
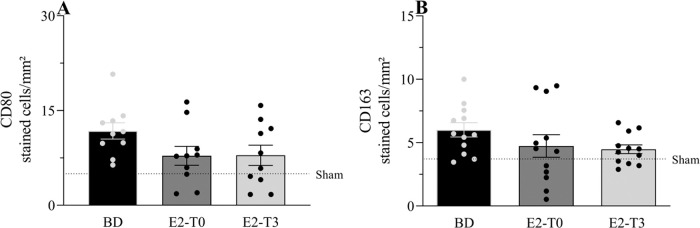
Lung protein expression of CD80 (A) and CD163 (B). Female rats were separated in groups: sham animals as control, BD animals subjected to brain death (BD), E2-T0 animals subjected to BD and treated with 17β-estradiol (E2) starting after BD confirmation, and E2-T3 animals subjected to BD and treated with E2 3h after BD confirmation. Data are expressed as mean±SEM from 5 photos/sample, 2 samples/animal, 5-6 animals/group. Kruskal-Wallis *p*=0.1534 (A) and *p*=0.1228 (B).

Immunohistochemical analysis of CD80 and CD163, markers for macrophage presence, is shown in Figure 4A and B, respectively. The numbers of CD80- and CD163-positive macrophages, which are highly present in lungs after BD, were not altered by the estradiol treatment.

### Long-lasting lung release of inflammatory mediators

**Figure 5:**
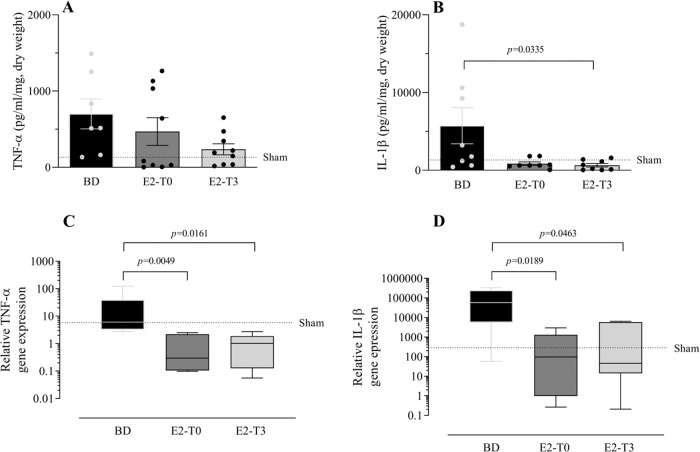
TNF-α and IL-1β levels in the lung culture medium (A, B) and lung relative gene expression (C, D). Female rats were separated into groups: sham animals as controls, BD animals subjected to brain death (BD), E2-T0 animals subjected to BD, and treated with 17β-estradiol (E2) starting after BD confirmation, and E2-T3 animals subjected to BD and treated with E2 3h after BD confirmation. (A, B) Data are expressed as mean±SEM from to 7-9 animals. (C, D) Data are expressed as median and 95% percentile interval from to 5-6 animals. Kruskal-Wallis *p*=0.1888 (A), *p*=0.0483 (B), *p*=0.005 (C) and *p*=0.0115 (D).

**Figure 6:**
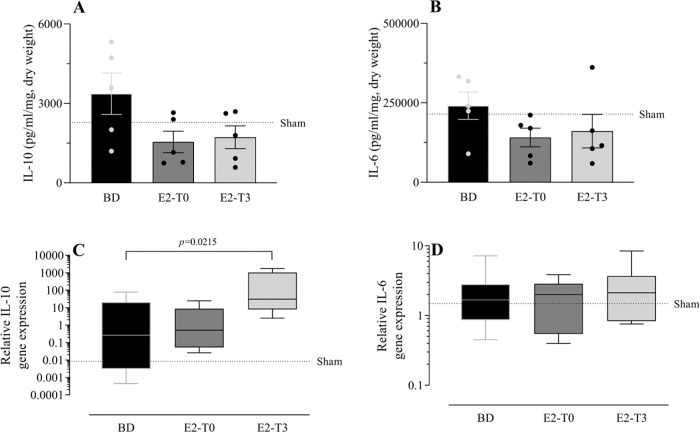
L-10 and IL-6 concentration in lung culture medium (A, B) and lung gene expression (C, D). Female rats were separated in groups: *sham* animals as control, *BD* animals subjected to brain death (BD), *E2-T0* animals subjected to BD and treated with 17β-estradiol (E2) starting after BD confirmation, and *E2-T3* animals subjected to BD and treated with E2 3h after BD confirmation. (A, B) Data are expressed as mean±SEM from 5 animals. (C, D) Data expressed as median and 95% percentile interval from 5-6 animals (C) and 7 animals (D). Kruskal-Wallis *p*=0.1449 (A), *p*=0.2391 (B), *p*=0.0247 (C) and *p*=0.8071 (D).

To study the effect of E2 on the long-lasting release of inflammatory mediators by the lungs after BD, we measured TNF-α, IL-1β, IL-10, and IL-6 in the lung culture medium (24h). In parallel, their gene expression was also quantified. Despite the reduction in TNF-α gene expression (Figure 5C) observed in the lungs of treated animals in both groups, its release by the lungs was lower only in the E2-T3 group (Figure 5A). Conversely, IL-1β content in the medium of the BD group lungs (Figure 5B) and tissue gene expression (Figure 5D) were reduced in both estradiol-treated groups. E2 treatment also tended to reduce IL-10 and IL-6 levels in the lung culture medium of animals with BD, but no significant differences were found (Figure 6A, B). In gene expression (Figure 6C, D), E2 induced an IL-10 increase in the E2-T3 group.

### Gene and protein analysis of caspase-3

**Figure 7:**
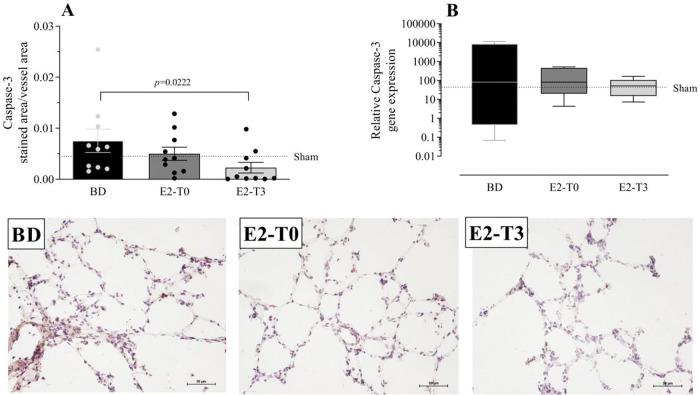
Lung gene (A) and protein (B) expression of caspase-3 with photomicrography of the immunohistochemical reaction. Female rats were separated in groups: *sham* animals as control, *BD* animals subjected to brain death (BD), *E2-T0* animals subjected to BD and treated with 17β-estradiol (E2) starting after BD confirmation, and *E2-T3* animals subjected to BD and treated with E2 3h after BD confirmation. (A) Data are expressed as mean±SEM from 5 photos/sample, 2 samples/animal, 5 animals/group. (B) Data expressed as median and 95% percentile interval from 6 animals. Kruskal-Wallis *p*=0.0328 (A) and *p*=0.7793 (B).

To analyze the effects of BD on apoptosis, we quantified the gene and protein expression of caspase-3 in the lung tissue (Figure 7A, B). Data showed that caspase-3 protein expression after BD model was reduced in the E2-T3 group. In relation to gene expression, we observed similar expression in all studied groups.

## DISCUSSION

The lung is one of the organs most vulnerable to the effects of BD, presenting higher rejection than other organs after transplant, since BD affects lung tissue homeostasis and triggers a persistent inflammatory process (3,4). As reported previously, the present BD experimental model results in lung injury, characterized by leukocyte infiltration and inflammatory mediators release in comparison to sham operated animals (15). In this study, our main focus was to determine whether estradiol treatment could influence the long-term inflammatory response to BD in the lungs. Therefore, it is important to mention the role of donor sex related to the risk of mortality after lung transplant, which is reported to be higher when lungs from female donors are transplanted in male recipients (7,8). Indeed, an experimental study identified donor sex as an important factor in lung graft quality, showing that females have more severe lung injury than males after BD, which has been associated with the reduction of female sex hormones. Female sex hormones have been proven to protect the lungs from injury after trauma events (5,6). Particularly, estradiol has shown short-term effects in controlling lung inflammation after BD (15). Thus, we analyzed aspects of the immune response known to be relevant to lung transplant success, such as complement system activity, macrophage presence, apoptosis, and lung release of inflammatory mediators. We found that estradiol reduced lung and systemic C3 and the IL-1β and TNF-α concentrations in lung cultures, showing a reduction in the long-lasting release of inflammatory mediators.

The complement system is an important part of the immune response, generating effector molecules that can potentially cause tissue injury, autoimmunity disease, alloreactivity, and transplant rejection (16). We have to consider that hormonally active females have lower complement system activity, when compared to males, and restricted terminal pathway activity, resulting in the inability to promote inflammation through the membrane attack complex (17,18). In our study, we found that estradiol could reduce complement activity until the C3 level, which is explained by the existence of an estradiol-responsive element in the promoter region (19). A lower complement system activity will increase lung transplant success (12,13). Indeed, by reducing C3, estradiol may inhibit actions of the active fragments such as C3a and C5a, which cause tissue damage by microvascular changes in flow and permeability, leukocyte extravasation, and migration (20). They would specifically attract and activate neutrophils and macrophages, releasing more complement fragments as well as other cytokines and chemokines (21).

In the lung, alveolar macrophages are the first line of defense, recognizing danger signals and attracting an influx of neutrophils and monocytes that quickly derive into inflammatory macrophages, amplifying the response. If the inflammatory stimuli continue, inflammation cannot be resolved and may cause tissue damage (22). Our data indicate an apparent increase in macrophages in the lung tissue caused by BD. Zhao et al. (23) showed that alveolar macrophages are essential for acute damage in the lung after ischemia and reperfusion, whereas neutrophils have a main role in long-term injury. In a previous study from our laboratory with female rats, we showed some cellular mechanisms by which estradiol controlled the inflammatory process that leads to lung injury after 6h in the BD model, especially by reducing leukocyte infiltration (15). However, we found that estradiol may exert a more prolonged effect on macrophage activation and recruited neutrophils. The concentration of mediators released in the lung culture, independent of further stimuli from the system and the expression of their genes, was reduced by E2. Considering how the BD inflammatory response was altered by an estradiol infusion to the donor suggests a reduction in the graft’s long-lasting inflammatory response.

In acute lung injury, TNF-α and IL-1β are considered “early response cytokines,” produced by alveolar macrophages through the activation of nuclear factor kappa B (24). They increase adhesion molecules and chemokine release, attracting more leukocytes. In the later phase of inflammation, neutrophils and macrophages produce superoxide anion and matrix metalloproteinases 2 and 9, causing damage to basal membranes and epithelial cells in the lung (3). These cells play an obligatory role in the persistence of the inflammatory response, leading to chronic inflammation (25). A previous study showed that estradiol reduces metalloproteinase activity, intercellular adhesion molecule 1 expression, and leukocyte infiltration (15). In this context, if estradiol is able to reduce the future release of TNF-α and IL-1β by resident and infiltrated cells, it would affect the long-term inflammatory response. In light of our results, we infer that estradiol plays a role in controlling lung inflammation by controlling leukocyte release of inflammatory mediators, such as IL-1 β and complement fragments, resulting in better lung quality and influencing the transplantation outcome.

Previously, we also reported that estradiol is able to reduce caspase-3 expression and increase BCL-2, an anti-apoptotic protein in the heart tissue of brain dead females (26). Our data showed that BD-induced lung apoptosis was regulated by estradiol treatment, as evidenced by the lower expression of caspase-3. Activation of caspase-3, a major executioner of caspase in the apoptosis process, signifies cell death. Therefore, a treatment that inhibits caspase could reduce lung injury (27,28). Notably, the complement system could also have a role in caspase-3 expression. A study by Hu et al. (29) identified that complement activity can lead to apoptosis of alveolar macrophages, contributing to the development of acute lung injury. We suggest that estradiol could influence apoptosis not only by reducing complement activity, but also by reducing the release of TNF-α, which is known to induce cell death signaling through caspase-3 activation, the extrinsic pathway (30).

We understand that female sex hormones play an important role in BD-induced short- and long-term lung inflammation and female donor treatment with estradiol could regulate this response. The results presented here strengthen the possibility of estradiol as a donor therapeutic treatment, resulting in the improvement of lung grafts. We suggest that further research on estradiol as a therapy for controlling lung graft immune response after transplant is necessary as well as its application in graft preservation maneuvers.

## AUTHOR CONTRIBUTIONS

Ricado-da-Silva FY and Armistrong-Jr performed the study, analyzed data and wrote the manuscript. Vidal-dos-Santos M, Correia CJ, Coutinho e Silva RS and Anunciação LF contributed obtaining and analyzing data. Breithaupt-Faloppa AC designed the study, analyzed data and wrote the manuscript. Leuvenink HGD and Moreira LFP analyzed data and wrote the manuscript.

## Figures and Tables

**Table 1:** Primer sequence used for real-time polymerase chain reaction (PCR) analysis.

Real-time PCR SYBR^®^Green		
β-actin	RN b-act fw	5′-GGAAATCGTGCGTGACATTAAA-3′
	RN b-act rv	5′GCGGCAGGGCCATCTC-3′
C3	RN C3 fw	5′-CAGCCTGAATGAACGACTAGACA-3′
	RN C3 rv	5′-TCAAAATCATCCGACAGCTCTATC-3′
C3aR	RN C3AR fw	5′-CCTAAGCAGATGTTCTGAGGTGAA-3′
	RN C3AR rv	5′-AGGGTGGCCAGGCTGTCTA-3′
DAF	RN DAF fw	5′-TGTCATCGTCTTGAAGGTGTGCTA-3′
	RN DAF rv	5′-TATGCATTGAAAAGACCATTCCAGA-3′
TNF-α	RN TNFa fw	5′-AGGCTGTCGCTACATCACTGAA-3′
	RN TNFa rv	5′-TGACCCGTAGGGCGATTACA-3′
IL-1β	RN IL-1B fw	5′-CAGCAATGGTCGGGACATAGTT-3′
	RN IL-1B rv	5′-GCATTAGGAATAGTGCAGCCATCT-3′
IL-10	RN IL-10 fw	5′-GCAACAGCTCAGCGCATCT-3′
	RN IL-10 rv	5′-ACAAACTGGTCACAGCTTTCGA-3′
IL-6	RN IL-6 fw	5′-CAACTTCCAATGCTCTCCTAATG-3′
	RN IL-6 rv	5′-TTCAAGTGCTTTCAAGAGTTGGAT-3′
Caspase-3	RN CASP-3 fw	5′-GCATGCCAGAAGATACCAGTGG-3′
	RN CASP-3 rv	5′-AGTTTCAGCATGGCGCAAA-3′

DAF, decay-accelerating factor; TNF, tumor necrosis factor; IL, interleukin.
